# *In silico* Screening of Natural Phytocompounds Towards Identification of Potential Lead Compounds to Treat COVID-19

**DOI:** 10.3389/fmolb.2021.637122

**Published:** 2021-07-05

**Authors:** Muthumanickam Sankar, Balajee Ramachandran, Boomi Pandi, Nachiappan Mutharasappan, Vidhyavathi Ramasamy, Poorani Gurumallesh Prabu, Gowrishankar Shanmugaraj, Yao Wang, Brintha Muniyandai, Subaskumar Rathinasamy, Balakumar Chandrasekaran, Mohammad F. Bayan, Jeyakanthan Jeyaraman, Gurumallesh Prabu Halliah, Solomon King Ebenezer

**Affiliations:** ^1^Department of Bioinformatics, Alagappa University, Karaikudi, India; ^2^Kumaraguru College of Technology, Coimbatore, India; ^3^Department of Biotechnology, Alagappa University, Karaikudi, India; ^4^Department of Biotechnology, School of Life Science, Anyang Institute of Technology, Anyang, China; ^5^Department of Animal Health and Management, Science Campus, Alagappa University, Karaikudi, India; ^6^Sri Ramakrishna College of Arts and Science, Bharathiar University, Coimbatore, India; ^7^Faculty of Pharmacy, Philadelphia University-Jordan, Philadelphia University, Jordan; ^8^Department of Industrial Chemistry, School of Chemical Sciences, Alagappa University, Karaikudi, India; ^9^Bioscience Research Foundation, Chennai, India

**Keywords:** COVID-19, main protease, molecular dynamics simulation, natural medicinal plants, S-ACE2, structure-based virtual screening

## Abstract

COVID-19 is one of the members of the coronavirus family that can easily assail humans. As of now, 10 million people are infected and above two million people have died from COVID-19 globally. Over the past year, several researchers have made essential advances in discovering potential drugs. Up to now, no efficient drugs are available on the market. The present study aims to identify the potent phytocompounds from different medicinal plants (*Zingiber officinale*, *Cuminum cyminum*, *Piper nigrum*, *Curcuma longa*, and *Allium sativum*). In total, 227 phytocompounds were identified and screened against the proteins S-ACE2 and M^*pro*^ through structure-based virtual screening approaches. Based on the binding affinity score, 30 active phytocompounds were selected. Amongst, the binding affinity for beta-sitosterol and beta-elemene against S-ACE2 showed −12.0 and −10.9 kcal/mol, respectively. Meanwhile, the binding affinity for beta-sitosterol and beta-chlorogenin against M^*pro*^ was found to be −9.7 and −8.4 kcal/mol, respectively. Further, the selected compounds proceeded with molecular dynamics simulation, prime MM-GBSA analysis, and ADME/T property checks to understand the stability, interaction, conformational changes, binding free energy, and pharmaceutical relevant parameters. Moreover, the hotspot residues such as Lys31 and Lys353 for S-ACE2 and catalytic dyad His41 and Cys145 for M^*pro*^ were actively involved in the inhibition of viral entry. From the in silico analyses, we anticipate that this work could be valuable to ongoing novel drug discovery with potential treatment for COVID-19.

## Introduction

The newly emerged pandemic disease of COVID-19 was first identified in Wuhan market, China in December 2019 (Ferraz et al., 2020; Qi et al., 2020; Shyr et al., 2020). At the same time, a group of people were confirmed as new cases and subsequently died from COVID-19 (Arul Murugan et al., 2020; Walls et al., 2020). The precautions provided by the Chinese government such as intense quarantine, wearing masks, and social distance helped them control the rising rate of new cases (Cheng et al., 2020). Even with these precautions, COVID-19 spread throughout the world (Battisti et al., 2020). Unluckily, as of February 2021 over 10 million people were said to be infected and over two million people had died. Despite the wide availability of antiviral drugs, an appropriate medicine has not yet been found. Thus, the mortality and morbidity (Touret et al., 2020) rates continuously increase every day. Several researchers are currently trying to develop novel therapeutic drugs against COVID-19. Due to the shortcomings of existing drugs, they are not potent against COVID-19. Therefore, an alternative safe and effective antiviral therapy is urgently needed. In order to overcome any shortcomings, plant-based phytocompounds play an important role in treating COVID-19 (Benarba and Pandiella, 2020). Indian medicinal plants are rich in the source of phytocompounds that exhibit several biological properties including antiviral and immunomodulatory activities (Mukhtar et al., 2008; Chirumbolo, 2012). Arul Murugan et al. (2020) reported that the phytocompounds may exhibit antiviral properties against COVID-19. Moreover, the phytocompounds from various medicinal plants may help to boost the immune response and fight pathogens to combat viral disease (Babich et al., 2020; Ben-Shabat et al., 2020; Khanna et al., 2020).

A computational method is a reliable approach to identify active phytocompounds from various databases. This strategy is currently employed in drug discovery research (Wang, 2020). Recently, we have reported on the promising phytocompounds identified from different plant sources through the computational approaches for the treatment of COVID-19 (Gowrishankar et al., 2021). In the drug discovery process with the help of a computational approach, the selection of structural proteins is one of the major tasks. Two proteins namely S-ACE2 and M^*pro*^ have been taken for the present study based on the higher resolution. SARS-CoV-2 contains a spike (S) protein that helps viral entry into the host cell and releases virus particles by attaching to a cell surface receptor called human angiotensin-converting enzyme 2 (ACE2) which facilitates viral transcription and replication (Schoeman and Fielding, 2019; Whisenant and Burgess, 2019; Cavasotto and Di Filippo, 2020). It is one of the most common therapeutic drug targets because of its prominent role in host cell binding (Tariq et al., 2020). The spike protein of SARS-CoV-2 bound with ACE2 has a higher binding affinity than that of SARS-CoV and ACE2 (Baig et al., 2020). The crystal structure of the S-ACE2 protein contains 603 amino acid residues with a resolution of 2.5Å. Moreover, this structure may be used to investigate the mechanism and binding strength of the viral complexes (Bai and Warshel, 2020). The main protease (M^*pro*^) is the most attractive drug target to combat COVID-19 that can be correlated with viral replication and transcription of the virus life cycle (Huynh et al., 2020; Meyer-Almes, 2020). The M^*pro*^ is responsible for the viral polyprotein proteolytic process and viral genome replication and transcription. Hence it is considered as an attractive drug target against COVID-19 (Gao et al., 2020; Cavasotto et al., 2021). The M^*pro*^ has a 1.3Å resolution crystal structure and a sequence similarity of 96% when compared to SARS-CoV. Several computational studies have been performed to identify a potent drug target against COVID-19, they suggested that the M^*pro*^ could be a significant drug target based on its binding mechanism and stability (Linh Nguyen et al., 2020).

In the present study, we selected five medicinal plants: *Zingiber officinale* (ginger), *Cuminum cyminum* (cumin), *Piper nigrum* (black pepper), *Curcuma longa* (turmeric), and *Allium sativum* (garlic) based on their different biological applications which include antioxidant, anti-inflammatory, anti-stress, and anti-cancer properties, relief from cardiovascular diseases and respiratory congestion, and anti-diabetic, immune-modulator, anti-microbial, anti-platelet, and cyclo-oxygenase-I (COX-1) inhibitory properties (Rajagopal et al., 2020). From the plants, a total of 227 phytocompounds **(33 for *Zingiber officinale*; 45 for *Cuminum cyminum*; 42 for *Piper nigrum*; 54 for *Curcuma longa*, and 54 for *Allium sativum*)** were screened against various SARS-CoV-2 protein targets (S-ACE2 and M^*pro*^ protein). Among them, the top binding affinity phytocompounds such as beta-sitosterol, beta-elemene and beta-chlorogenin were identified. The selected compounds were taken for further assessment using molecular dynamics simulation, ADME/T, and drug likeness properties.

## Materials and Methods

The X-ray crystal structures of S-ACE2 with a resolution of 2.45 Å (PDB ID-6M0J) and M^*pro*^ with a resolution of 1.31 Å (PDB ID- 5R82) were retrieved from the Protein Data Bank (Chikhale et al., 2020). The obtained crystal structures were prepared individually with the following steps: water molecules and bound ions were removed, missing hydrogen atoms were added, and Kollman charges were allocated. Then, the energy of the protein structure was minimized using GROMOS 43b1 force field with the help of Swiss-Pdb Viewer. Finally, the structures were saved in the.pdbqt format for molecular docking.

The five selected Indian medicinal plants (*Zingiber officinale*, *Cuminum cyminum*, *Piper nigrum*, *Curcuma longa*, and *Allium sativum*) contain 227 phytocompounds (Supplementary Table 1), which were retrieved from the Pubchem database^1^ and downloaded in the SDF file format (Adejoro et al., 2020).

### Structure-Based Virtual Screening

Structure-based virtual screening (SBVS) plays a vital role in identifying essential phytocompounds from different Indian natural medicinal plants for the development of drug-like compounds. The Vina wizard of the PyRx 8.0 virtual screening tool was employed to screen the phytocompounds against the S-ACE2 and M^*pro*^ target proteins (Rowaiye et al., 2020). All the phytocompounds were energy-minimized using the steepest descent gradient algorithm with an MMFF94 force field using the OpenBabel program. Subsequently, the prepared structures were converted into the.pdbqt file format. The 3D grid maps were generated to cover the active site of the target proteins.

Among the 227 phytocompounds, 30 phytocompounds were selected based on their top binding affinity against S-ACE2 and M^*pro*^ proteins. The selected 30 phytocompounds are highlighted in Supplementary Table 1. From the above-mentioned 30 compounds, the top three phytocompounds (beta-elemene, beta-sitosterol, and beta-chlorogenin) from each plant which exhibited higher binding affinities against S-ACE2 and M^*pro*^ were selected for further assessment. The interaction profiles of protein-ligand complexes were viewed using the Discovery Studio Visualizer 2019 (Halder et al., 2019). The top three compounds were further validated with AutoDock 4.2 and AutoDock Vina tools to compare the binding affinity.

### Prime MM-GBSA

Prime MM-GBSA (Molecular Mechanics, the Generalized Born model, and Solvent Accessibility) analysis for S-ACE2 with beta-elemene and beta-sitosterol; M^*pro*^ with beta-sitosterol and beta-chlorogenin complexes was performed to calculate the binding free energies using Schrodinger, LLC, NY (Muthumanickam et al., 2020; Ramachandran et al., 2020). The following equation was used to enumerate the binding free energy.

ΔG=b⁢i⁢n⁢dG-c⁢o⁢m⁢p⁢l⁢e⁢x(G+p⁢r⁢o⁢t⁢e⁢i⁢nG)l⁢i⁢g⁢a⁢n⁢d

The G_*complex*_ indicates complex energy, G_*protein*_ indicates the receptor energy, and G_*ligand*_ indicates the unbound ligand energy.

### ADME/T (Absorption, Distribution, Metabolism, Elimination, and Toxicity) Prediction

After the SBVS of the 227 phytocompounds against target proteins, based on the binding energy values, the phytocompounds, beta-elemene, beta-sitosterol, and beta-chlorogenin, were considered for ADME/T properties using the pkCSM server^2^ to predict their pharmacokinetic properties (Guo et al., 2016).

### Molecular Dynamics Simulation and Post-MM/GBSA Analyses

Furthermore, to evaluate the structural stability of the S-ACE2 with beta-elemene and beta-sitosterol; M^*pro*^ with beta-sitosterol and beta-chlorogenin complexes, Molecular dynamics simulation (MDS) was performed using the GROMACS package with a GROMOS96 43a1 force field (Chandra Babu et al., 2017). The topology and parameters for the ligands were obtained from the PRODRG server^3^. The protein-ligand complexes were equilibrated under cubic periodic boundary conditions with the dimensions of 10 nm × 10 nm × 10 nm and solvated with an explicit SPC (simple point charge) water model. The proper counter ions Na^+^Cl^–^ were added to neutralize the system. The energy minimization steps were performed using the steepest descent gradient algorithm to remove the weak van der Waals contacts. The NPT and NVT ensembles were employed for 50,000 steps in 100 ps, respectively. Then a constant temperature was maintained at 300 K with a coupling time of 0.1 ps. The constant pressure was also maintained with 1 bar for 100 ps. Further, the MDS was carried out for 100 ns for all the protein-ligand complexes. The trajectories were analyzed for docked complexes to calculate the root mean square deviation (RMSD), root mean square fluctuation (RMSF), and hydrogen bond using scripts included in the GROMACS package. Each trajectory was graphically visualized using the XMGRACE 2D plotting tool. In addition, the post MM/GBSA analysis was performed to calculate the binding energy using the prime MM/GBSA module in Schrodinger.

## Results and Discussion

### Structure-Based Virtual Screening

Firstly, the co-crystallized compound and known binders such as Lopinavir and Rilapladib were re-docked against S-ACE2 and M^*pro*^. The co-crystallized compound of S-ACE2 showed a binding affinity of −6.9 kcal/mol, and a binding affinity of −7.0 kcal/mol for M^*pro*^. The known binder result reveals that Lopinavir and Rilapladib against S-ACE2 showed −5.4 and −6.0 kcal/mol, respectively. On the other hand, Lopinavir and Rilapladib against M^*pro*^ showed −7.5 and −6.7 kcal/mol, respectively.

Based on the binding affinity of the above reported compounds, our study aimed to investigate the potential therapeutic drug candidates to combat COVID-19. The identified 227 phytocompounds from five different natural medicinal plants were screened against target proteins such as S-ACE2 and M^*pro*^. Table 1 represents the binding affinity of the top phytocompounds against S-ACE2 and M^*pro*^.

**TABLE 1:** Binding affinity for the top phytoconstituents against S-ACE2 and M^*pro*^ proteins.

**S-ACE2 (PDB ID—6M0J)**									
***Zingiber officinale*—*ginger***		***Cuminum cyminum*—*cumin***		***Piper nigrum*—*black pepper***		***Curcuma longa*—*turmeric***		***Allium sativum*—*garlic***	
**Compound name**	**Binding affinity (Kcal/mol)**	**Compound name**	**Binding affinity (Kcal/mol)**	**Compound name**	**Binding affinity (Kcal/mol)**	**Compound name**	**Binding affinity (Kcal/mol)**	**Compound name**	**Binding affinity (Kcal/mol)**
Alpha-selinene	−10.9	Beta-elemene	−10.9	Beta-selinene	−7.8	Beta-sitosterol	−12.0	Saponin	−10.6
Beta-sitosterol	−12.0	Alpha-selinene	−10.9	Beta-elemene	−10.9	Cyclocurcumin	−9.1	Beta-tocopherol	−10.5
Beta-elemene	−10.9	Beta-sitosterol	−12.0	Beta-sitosterol	−12.0	Demethoxycurcumin	−8.9	Beta-chlorogenin	−8.4
**M^*pro*^ (PDB ID—5R82)**									
Beta-sitosterol	−9.7	Beta-sitosterol	−9.7	Beta-sitosterol	−9.7	Beta-sitosterol	−9.7	Beta-chlorogenin	−8.4
Kaempferol	−7.5	Apigetrin	−8.0	Alpha-tocopherol	−8.0	Alpha-tocopherol	−8.0	Apigenin	−7.5
Alpha-selinene	−7.0	Alpha-selinene	−7.2	Kaempferol	−7.5	Riboflavin	−7.2	Kaempferol	−7.5

Based on their higher binding affinity, beta-sitosterol (−12.0 kcal/mol) and beta-elemene (−10.9 kcal/mol) against S-ACE2 and beta-sitosterol (−9.7 kcal/mol) and beta-chlorogenin (−8.4 kcal/mol) against M^*pro*^ were taken for further studies. Furthermore, the AutoDock and AutoDock Vina tools provided a close binding affinity score against S-ACE2 and M^*pro*^ when compared with the PyRx tool (Table 2).

**TABLE 2:** Docking score for the top phytoconstituents against S-ACE2 and M^*pro*^ proteins.

**Compound name**	**AutoDock (Kcal/mol)**	**AutoDock Vina (Kcal/mol)**
**S-ACE2 (6M0J)**		
Beta-sitosterol	−12.3	−11.09
Beta-elemene	−9.8	−9.3
**M^*pro*^ (5R82)**		
Beta-sitosterol	−10.01	−8.9
Beta-chlorogenin	−8.8	−7.1

### The Binding Affinities of the Phytocompounds Into the S-ACE2 Active Site

**FIGURE 1:**
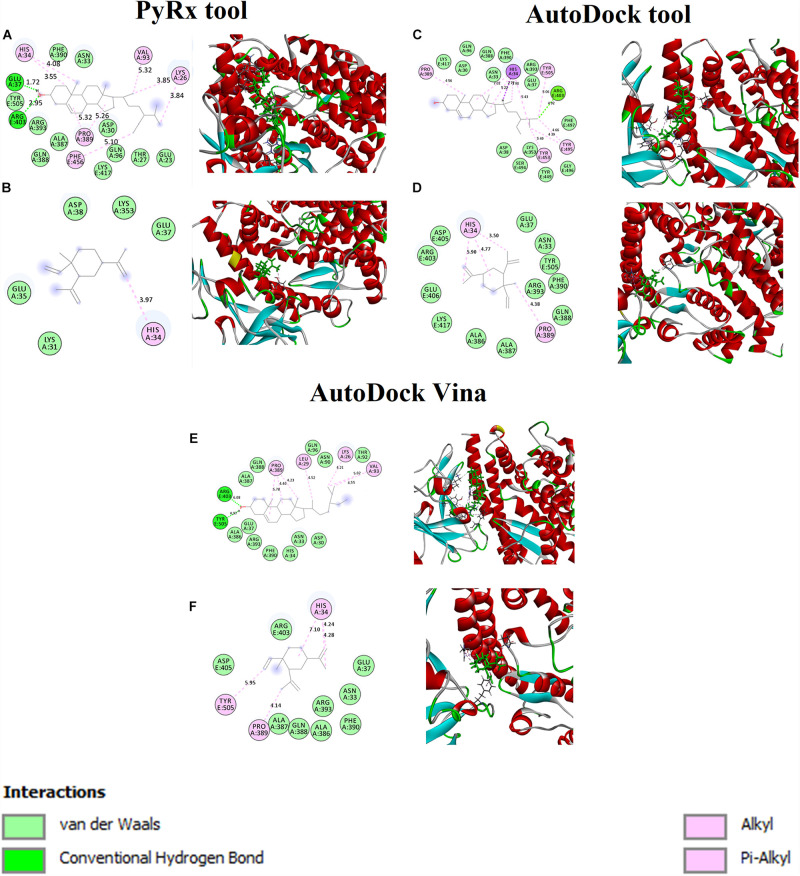
Binding affinity and amino acid interactions of phytocompounds. **(A,C,E)** S-ACE2 complexed with beta-sitosterol and **(B,D,F)** S-ACE2 complexed with beta-elemene.

The binding affinity for S-ACE2 with the beta-elemene and beta-sitosterol complexes were analyzed and shown in Figures 1A,B. Figure 1A displays the hydrogen bond, hydrophobic, and van der Waals interactions. Amino acid residues such as Glu37 and Arg403 actively participated in hydrogen bond interactions. Residues like Lys26, His34, Val93, Pro389, and Phe456 were actively involved with hydrophobic interactions. In van der Waals interactions, the Glu23, Thr27, Asp30, Asn33, Gln96, Ala387, Gln388, Phe390, Arg393, and Tyr505 residues actively interacted with beta-sitosterol. Polar amino acid residues such as Thr27, Gln96, and Tyr505 contributed to the interactions. Figure 1B reveals the hydrophobic and van der Waals interactions. Hydrophobic interactions involved the His34 residue. The van der Waals interaction was influenced by the Glu35, Glu37m and Asp38 residues along with hotspot residues **Lys31** and **Lys353**. A recent study reports that the beta-elemene phytocompound can interact with the hotspot residues of **Lys31** and **Lys353** which play a crucial role for the inhibition of viral entry (Choudhary et al., 2020). The interacting residues of these complexes are shown in Table 3.

**TABLE 3:** Interaction residues of beta-sitosterol and beta-elemene with S-ACE2 complexes.

**Phytocompound name**	**H-bond**	**Hydrophobic interaction**	**Van der Waals interaction**
Beta-sitosterol	Glu37 and Arg403	Lys26, His34, Val93, Pro389, and Phe456	Glu23, Thr27, Asp30, Asn33, Gln96, Ala387, Gln388, Phe390, Arg393, and Tyr505
Beta-elemene	-	His34	Glu35, Glu37, Asp38, **Lys31, and Lys353**

*The bolded Lys31 and Lys353 are the important hotspot residues for S-ACE2, and His41 and Cys145 are the essential catalytic dyads for M^pro^. They are actively involved in the inhibition of viral entry.*

Furthermore, the beta-sitosterol and beta-elemene were docked with S-ACE2 using the AutoDock (Figures 1C,D) and AutoDock Vina (Figures 1E,F) tools. The results showed that both the beta-sitosterol and beta-elemene complexes had similar binding affinity and interactions.

Generally, the mediating region of the S-protein of SARS-CoV-2 is highly responsible for contact with the ACE2 receptor through the receptor binding domain (RBD) which is present in host cells. SARS-CoV-2 enters into human cells through the ACE2 receptor, where targeting the receptor provides more insights on controlling SARS-CoV-2 infections. Thus, structure-based drug design (SBDD) approaches have been employed to identify the potential phytocompounds which could prevent the binding with the SARS-CoV-2 ACE2 receptor (Choudhary et al., 2020). According to the structure-based virtual screening (SBVS) results, this study provides selected phytocompounds may be potential inhibitors against SARS-CoV-2.

### The Binding Affinities of the Phytocompounds Into Catalytic Dyad of M^*pro*^

**FIGURE 2:**
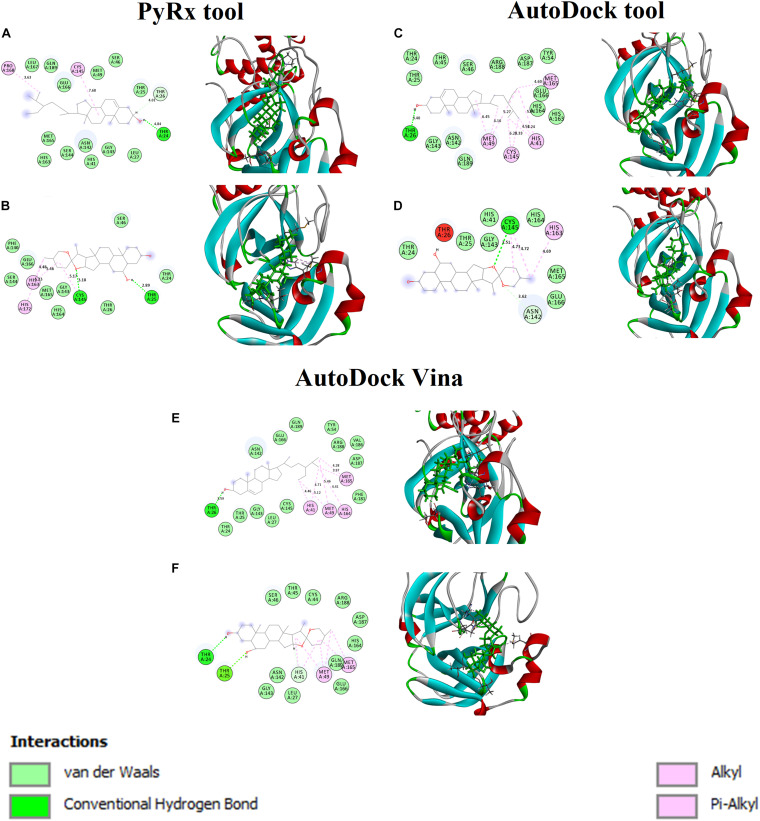
Binding affinity and amino acid interactions of phytocompounds. **(A,C,E)** M^*pro*^ complexed with beta-sitosterol and **(B,D,F)** M^*pro*^ complexed with beta-chlorogenin.

Figures 2A,B shows the binding affinity of the M^*pro*^ complexed with beta-sitosterol and beta-chlorogenin. As shown in Figure 2A, beta-sitosterol had one hydrogen bond interaction with Thr24 and two hydrophobic interactions with residues **Cys145** and Phe168. Also, the study observed that the van der Waals interactions involved the following residues: Thr25, Thr26, Leu27, **His41**, Ser46, Met49, Asn142, **Gly143**, Ser144, His163, Met165, Glu166, Leu167, and Gln189. As shown in Figure 2B, beta-chlorogenin formed two hydrogen bond interactions (Thr25 and **Cys145** residues) and three hydrophobic interactions (**Cys145**, His163, and His172 residues). Van der Waals interactions with the residues Thr24, Thr25, Ser46, Phe140, Gly143, Ser144, His164, and Met165 were noticed. Polar amino acids such as Thr25 and Cys145 were influenced. The interaction residues of these complexes are shown in Table 4.

**TABLE 4:** Interaction residues of beta-sitosterol and beta-chlorogenin with M^*pro*^ complexes.

**Phytocompound name**	**H-bond**	**Hydrophobic interaction**	**Van der Waals interaction**
Beta-sitosterol	Thr24 and Thr26	**Cys145 and** Pro168	Thr25, Leu27, **His41**, Ser46, Met49, Asn142, Gly143, Ser144, His163, Met165, Glu166, Leu167, and Gln189
Beta-chlorogenin	Thr25 and **Cys 145**	**Cys145**, His163, and His172	Thr24, Thr25, Ser46, Phe140, Gly143, Ser144, His164, and Met165

*The bolded Lys31 and Lys353 are the important hotspot residues for S-ACE2, and His41 and Cys145 are the essential catalytic dyads for M^pro^. They are actively involved in the inhibition of viral entry.*

In earlier reports, the catalytic dyad **Cys145** and **His41** residues of M^*pro*^ actively participated in the binding regions (Alexpandi et al., 2020; Bharadwaj et al., 2020). In our study, the aforementioned selected phytocompounds actively interacted with the binding pocket of the catalytic dyad (**Cys145** and **His41**) of the substrate binding domain of the M^*pro*^. The substantial binding affinity and formation of strong interactions between the above residues indicated the stability of the docked complexes.

The M_*pro*_ with beta-sitosterol and beta-chlorogenin complexes were further analyzed using AutoDock (Figures 2C,D) and AutoDock Vina (Figures 2E,F) tools. These showed similar binding affinity and interactions. Therefore, the molecular docking analysis suggested that beta-sitosterol and beta-chlorogenin play a vital role in replication and pathogenesis. Hence, it may inhibits COVID-19 M^*pro*^ enzyme activity which is taken further in molecular dynamics simulations.

### Binding Free Energy

To evaluate the binding free energy of the complexes, the MM/GBSA approach was employed (Table 5). The results revealed that S-ACE2 with the beta-elemene and beta-sitosterol complexes showed binding free energies of −28.87 and −26.17 kcal/mol, respectively. The binding free energy of M^*pro*^ with the beta-sitosterol and beta-chlorogenin complexes were found to be −33.21 and −10.59 kcal/mol, respectively. The overall binding free energies indicate that the lead molecules such as beta-elemene, beta-sitosterol, and beta-chlorogenin may strongly bind in the binding region of taken targets to inhibit enzymatic activity as well as the innate immune response of the human body to prevent COVID-19.

**TABLE 5:** Binding free energy of top phytocompounds against S-ACE2 and M^*pro*^.

**S.No**	**Compound name**	**ΔG_*bind*_**	**ΔG_*covalent*_**	**ΔG_*coulomb*_**	**ΔG_*HBond*_**	**ΔG_*Lipo*_**	**ΔG_*SolvGB*_**	**ΔG_*vdW*_**
**S-ACE2 (6M0J)**								
1	Beta-sitosterol	−28.87	2.95	−1.10	−1.18	−11.78	25.09	−33.04
2	Beta-elemene	−26.17	4.88	−1.54	−0.81	−12.01	28.59	−33.00
**M^*pro*^ (5R82)**								
3	Beta-sitosterol	−33.21	5.57	−5.28	−1.01	−15.17	21.19	−38.50
4	Beta-chlorogenin	−40.59	1.93	−9.35	−0.47	−15.47	17.90	−35.20

### ADME/T Properties

The efficiency of the therapeutic phytocompounds mainly depends on their bioactivity and pharmacokinetics properties. The ADME/T properties of beta-elemene, beta-sitosterol, and beta-chlorogenin were computed using the PkCSM web server, and are shown in Table 6. The selected phytocompounds showed an acceptable range including < 500 molecular weight, < 10 hydrogen bond acceptors, < 5 hydrogen bond donors, and logP values of < 5. Furthermore, the pharmacokinetic parameters such as human oral absorption, partition co-efficient (QPlog*P*o/w), and water solubility (QPlogS) values did not violate Lipinski’s RO5. Hence, the observed values for the selected phytocompounds can be considered as potent inhibitors for COVID-19.

**TABLE 6:** ADME/T properties of the top phytocompounds using the PkCSM web server.

**Properties**	**Beta-elemene**	**Beta-sitosterol**	**Beta-chlorogenin**
Molecular weight	204.357	414.718	432.645
TPSA	94.774	187.039	188.008
LogP	4.7472	8.0248	4.7646
H-bond acceptor	0	1	4
H-bond donor	0	1	2
**Absorption**			
Water solubility (log mol/L)	−6.43	−6.773	−5.213
Caco2 permeability (log Papp in10-6 cm/s)	1.41	1.201	1.263
Intestinal absorption (human) (% absorbed)	94.359	94.464	96.823
Skin permeability (log Kp)	−1.279	−2.783	−3.999
P-Glycoprotein substrate	No	No	Yes
P-Glycoprotein I inhibitor	No	Yes	Yes
P-Glycoprotein II inhibitor	No	Yes	Yes
**Distribution**			
VDss (human, log L/kg)	0.601	0.193	0.192
Fraction unbound (human) (Fu)	0.157	0	0.037
BBB permeability (logBB)	0.809	0.781	0.004
CNS permeability (log PS)	−1.714	−1.705	−1.592
**Metabolism**			
CYP2D6 substrate	No	No	No
CYP3A4 substrate	No	Yes	Yes
CYP1A2 inhibitor	No	No	No
CYP2C19 inhibitor	No	No	No
CYP2C9 inhibitor	No	No	No
CYP2D6 inhibitor	No	No	No
CYP3A4 inhibitor	No	No	No
**Excretion**			
Total clearance (log ml/min/kg)	0.251	0.628	0.346
Renal OCT2 substrate	No	No	Yes
**Toxicity**			
AMES toxicity	No	No	No
hERG I inhibitor	No	No	No
hERG II inhibitor	No	Yes	No
Hepatotoxicity	No	No	No
Skin sensitization	Yes	No	No

In addition, the phytocompounds displayed negative AMES toxicity and negative carcinogenicity which indicated that they are non-mutagenic and non-carcinogenic. From the results, it was noticed that the selected phytocompounds had an acceptable range except beta-sitosterol. It was because the LogP value was 8, hence beta-sitosterol had violated the drug-likeliness properties. Several reports suggested that the beta-sitosterol phytocompound possessed anti-HIV and anti-HBV (hepatitis B virus) activities (Tanaka et al., 2004; Parvez et al., 2019). In addition to this, beta-sitosterol has shown activity against SARS-CoV-2. An earlier study suggested that with the availability of beta-sitosterol in the drug industry, this molecule could be considered as a potential inhibitor of SARS-CoV-2 (Chowdhury, 2019; Parvez et al., 2019). In line with this, beta-sitosterol has been considered to have potential antiviral activity against COVID-19.

### Molecular Dynamics Simulations

**FIGURE 3:**
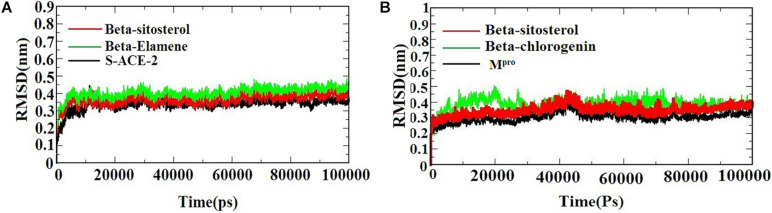
The RMSD of the protein-ligand complexes during the 1,000 ns MD simulations. **(A)** S-ACE2 with beta-sitosterol and beta-elemene complexes and **(B)** M^*pro*^ with beta-sitosterol and beta-chlorogenin complexes.

A molecular dynamics simulation was performed to evaluate the stability and conformational changes of the docked complex. Figures 3A,B) represents the MDS of S-ACE2 with the beta-sitosterol and beta-elemene complexes; M^*pro*^ with the beta-sitosterol and beta-chlorogenin complexes, respectively. To examine the degree of conformational changes in the protein (backbone) and docked complexes, RMSD was calculated for 100 ns simulation trajectories for each complex. The RMSD of the backbone of S-ACE2 showed stability throughout the simulation (indicated in black) by maintaining a peak of 0.3 nm. S-ACE2 with the beta-sitosterol (indicated in red) and beta-elemene (indicated in green) complexes also occurred in the acceptable range and no major fluctuations were observed when compared to the backbone (Figure 3A). The backbone atoms of M^*pro*^ (indicated in black) maintained a peak with equilibration ranges between 0.25 and 0.4 nm and showed better stability until the end of the simulation. On the other hand, the M^*pro*^ with beta-sistosterol showed a similar acceptable range from 0.3 to 0.45 nm (indicated in red). The beta-chlorogenin equilibrated at 5 ns, fluctuated < 0.1 nm (on an average), and showed constant peaks throughout the simulation (indicated in green). Hence, all the three converged at 40 ns and were constant at the end of the simulation (Figure 3B). RMSD of the complexes were calculated as a function of time period to evaluate the conformational stability of the phytocompounds (Selvaraj and Singh, 2014). The trajectory analysis revealed that the protein-ligand complexes were stable and active in dynamic movement after attaining the equilibrium state. From the obtained results, it was observed that the taken complexes were stable throughout the simulation.

**FIGURE 4:**
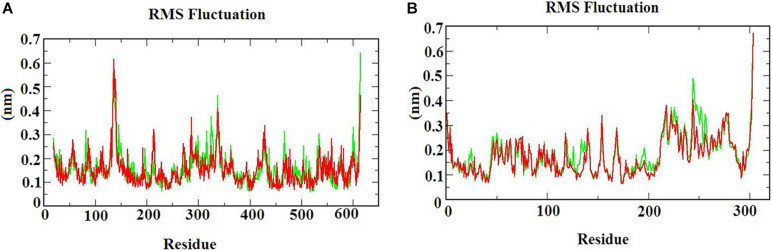
The RMSF of the protein-ligand complexes during the 100 ns MD simulations. **(A)** S-ACE2 with beta-sitosterol and beta-elemene complexes and **(B)** M^*pro*^ with beta-sitosterol and beta-chlorogenin complexes.

The RMSF graph provides information about the residual fluctuation of the protein-ligand complex. Figure 4 illustrates the RMSF of (A) S-ACE2 with beta-sitosterol and beta-elemene complexes; (B) M^*pro*^ with beta-sitosterol and beta-chlorogenin complexes for the period of 100 ns MD simulations. The average RMSF was maintained at 0.33 nm for S-ACE2 and 0.28 nm for M^*pro*^ complexes. For S-ACE2 with beta-sitosterol and beta-elemene complexes, the residues fluctuated between 100 and 120, 300, and 320. The following residues such as Asp615 (0.5 nm), Asp136 (0.6 nm), Asn338 (0.4 nm), and Val339 (0.4 nm) had a higher fluctuation with beta-sitosterol. Subsequently, the Asp615 (0.7 nm), Tyr613 (0.5 nm), Asn338 (0.4 nm), and Ala614 (0.4 nm) residues fluctuated in beta-elemene with the S-ACE2 complex. In the M^*pro*^ with beta-sitosterol and beta-chlorogenin complexes, fluctuations were observed between the amino acids 200 and 225. The M^*pro*^ with beta-sitosterol fluctuated for the following residues: Thr304 (0.6 nm), Phe223 (0.4 nm), Gln244 (0.4 nm), and Val303 (0.4 nm). The fluctuations for the M^*pro*^ with beta-chlorogenin complex were observed for residues Thr304 and Val303 (0.5 nm). The fluctuation of amino acids may be involved in the loop region which was monitored in the molecular dynamics simulations. From the outcome of the RMSF analysis, it was observed that the complexes were stable and the fluctuated residues did not have any impact on the protein-ligand complex.

**FIGURE 5:**
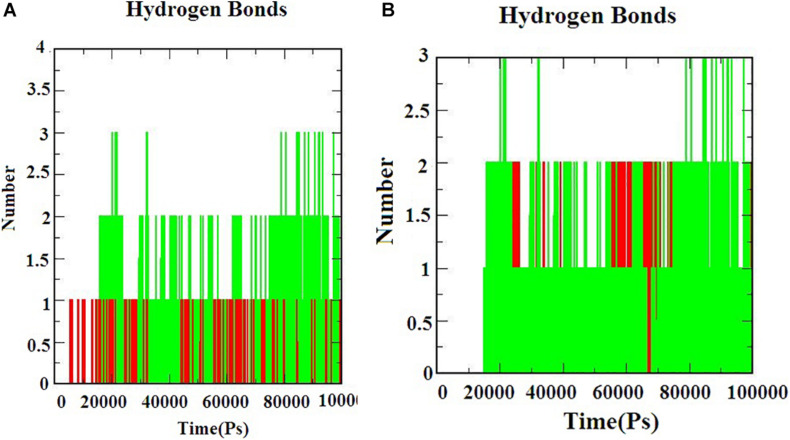
The H-bond interaction of the protein-ligand complexes during the 100 ns MD simulations. **(A)** Beta-sitosterol and beta-elemene complexed with S-ACE2 and **(B)** beta-sitosterol and beta-chlorogenin complexed with M^*pro*^.

Hydrogen bond interactions are the most important to determine the bonding strength between the protein-ligand complex. Figures 5A,B) indicates the number of hydrogen bonds formed during the simulation. The hydrogen bonds for S-ACE-2 with beta-sitosterol and beta-elemene complexes; M^*pro*^ with beta-sitosterol and beta-chlorogenin complexes increased during the simulations which indicated stable binding. Overall the simulation analysis indicated that no conformational changes were observed during the simulation. Therefore, the identified phytocompounds could be promising candidates for inhibiting S-ACE2 and M^*pro*^.

### Post MM/GBSA Analyses

The post MM/GBSA of the binding free energy calculation was analyzed with the generation of four-frame time intervals (25, 50, 75, and 100 ns) throughout the MDS as shown in Table 7. The results showed that all the complexes had better binding free energy at 100 ns when compared with the pre-MM/GBSA binding free energy.

**TABLE 7:** Post MM/GBSA of top phytocompounds against S-ACE2 and M^*pro*^.

**S.No**	**Compound name**	**ΔG_*bind*_ (25 ns)**	**ΔG_*bind*_ (50 ns)**	**ΔG_*bind*_ (75 ns)**	**ΔG_*bind*_ (100 ns)**
**S-ACE2 (6M0J)**					
1	Beta-sitosterol	−16. 09	−19.09	−23.96	−31.48
2	Beta-elemene	−12.24	−19.98	−21.90	−28.09
**M^*pro*^ (5R82)**					
3	Beta-sitosterol	−9.24	−16.55	−29.09.	−36.01
4	Beta-chlorogenin	−13.00	−20.24	−29.99	−45.77

## Conclusion

Traditionally, drugs from medicinal plant sources have been widely used to control disease. At this critical stage of the COVID-19 infection, an effective drug is urgently needed. Plants contain a rich source of phytocompounds which may be an effective approach to combat COVID-19. The present study revealed the better binding affinities of beta-sitosterol, beta-elemene, and beta-chlorogenin in the active site of the S-ACE2 and M^*pro*^ proteins. In addition, MDS revealed that the amino acid fluctuation may be involved in the loop region that exhibits stability throughout the simulation, and hydrogen bonds for all the complexes increased during the simulations. Furthermore, the post-MM/GBSA showed that all the complexes had better binding free energy at 100 ns when compared with pre-MM/GBSA binding free energy. Moreover, the ADME/T properties confirmed that the identified phytocompounds could be considered as promising drug-like compounds. The identified phytocompounds (beta-sitosterol, beta-elemene, and beta-chlorogenin) are publicly available which will facilitate the rapid development of suitable effective therapeutic candidates to control viral replication as well as the innate immune response to combat COVID-19. Therefore, the current study will be highly useful for further research to design specific drugs against COVID-19. In future, the identified phytocompounds will be extended through experimental studies to confirm their status as novel compounds against COVID-19x.

## Data Availability Statement

The original contributions presented in the study are included in the article/Supplementary Material, further inquiries can be directed to the corresponding author/s.

## Author Contributions

MS: investigation, methodology, and writing—original draft. BP: conceptualization, methodology, writing—review and editing, and supervision. NM: analyzing the result and discusses the manuscript. BR: discussing the *in silico* work and writing the manuscript. VR: editing the manuscript. PP: methodology design and editing. GS: developed the theoretical frame work. YW: review and editing, and designed the figure. BM: aided in interpreting the results and worked on the manuscript. BC: review and editing, and supervision. MB: review and editing the manuscript. SR: contributed to the interpretation of the results. JJ: contributed to the final version of the manuscript. GH: writing—review and editing, and supervision. SE: analyze the result and writing the manuscript. All authors contributed to the article and approved the submitted version.

## Conflict of Interest

The authors declare that the research was conducted in the absence of any commercial or financial relationships that could be construed as a potential conflict of interest.

## Footnotes

1 https://pubchem.ncbi.nlm.nih.gov/

2 http://biosig.unimelb.edu.au/pkcsm/

3 http://prodrg1.dyndns.org/
