# Supplementary material for: In silico Screening of Natural Phytocompounds Towards Identification of Potential Lead Compounds to Treat COVID-19
Source: Front Mol Biosci. 2021 Jul 5;8:637122. doi: 10.3389/fmolb.2021.637122 (PMC8288047; doi:10.3389/fmolb.2021.637122)
Supplement: Supplementary file 1 [file Table_1.DOCX]

**Supplementary table 1**

**Phytoconstituents available in the natural medicinal plants and Docking results**

| **S.No** | **Compound ID** | **Name of the phytoconstituents** | **Binding affinity**  **(kcal mol-1)** | |
| --- | --- | --- | --- | --- |
|  |  |  | **5R82(M^pro^)** | **6MOJ**  (S-ACE2) |
| ***1. Zingiber offinale*** | | | | |
| 1 | 44559528 | (S)-6-Gingerol | -5.1 | -6.8 |
| 2 | 3083834 | (+)-Alpha-curcumene | -5.7 | -6.7 |
| 3 | 7140311 | (+)-Angelicoidenol | -4.4 | -6.4 |
| 4 | 442484 | +)-Beta-phellandrene | -5.5 | -6.5 |
| 5 | 6552009 | (+)-Borneol | -5.7 | -6.7 |
| 6 | 2758 | 1,8-Cineole | -4.7 | -6.7 |
| 7 | 5318274 | [4]-Gingerdiol 3,5-diacetate | -5.2 | -6.2 |
| 8 | 14440539 | 10-Epizonarene | -5.2 | -5.2 |
| 9 | 10954686 | 10-Gingerdione | -5.1 | -5.1 |
| 10 | 442793 | 6-Gingerol | -5.4 | -5.9 |
| 11 | 6442612 | 10-shogaol | -6.7 | -6.7 |
| 12 | 86196540 | 12-Gingediol | -4.6 | -6.6 |
| 13 | 5317599 | 12-Gingerol | -5.3 | -7.5 |
| 14 | 46901319 | 4-Gingerol | -5.4 | -7.4 |
| 15 | 11127403 | Alpha-Zingiberene | -5.0 | -5.0 |
| 16 | 7462 | Alpha-Terpinene | -3.7 | -3.7 |
| 17 | 17100 | Alpha-Terpineol | -5.0 | -5.0 |
| 18 | 65575 | Alpha-Cedrol | -4.0 | -5.7 |
| 19 | 442355 | Alpha-Copaene | -4.9 | -5.9 |
| 20 | 442359 | Alpha-Cubebene | -4.6 | -4.6 |
| 21 | 92139 | Alpha-Curcumene | -5.4 | -5.4 |
| 22 | 5281516 | Alpha-Farnesene | -5.8 | -5.8 |
| 23 | 5280934 | Linolenic acid | -4.0 | -5.0 |
| 24 | 7460 | Alpha-Phellandrene | -5.9 | -4.9 |
| 25 | 6654 | Alpha-Pinene | -5.1 | -7.1 |
| **26** | **10856614** | **Alpha-Selinene** | **-7.0** | **-10.9** |
| **27** | **11127403** | **Alpha-Zingiberene** | **-6.9** | **-6.9** |
| **28** | **6918391** | **Beta-Elemene** | **-7.1** | **-10.9** |
| **29** | **222284** | **Beta-sitosterol** | **-9.7** | **-12.0** |
| **30** | **5280863** | **Kaempferol** | **-7.5** | **-7.5** |
| 31 | 853433 | Isoeugenol | -6.3 | -6.9 |
| 32 | 969516 | Curcumin | -6.2 | -5.9 |
| 33 | 22311 | Limonene | -4.4 | -5.4 |
| ***2. Cuminum Cyminum*** | | | | |
| 34 | 2758 | 1,8-Cineol | -4.7 | -6.7 |
| 35 | 4443160 | Alpha phellandrene | -5.9 | -4.9 |
| **36** | **6654** | **Alpha-pinene** | **-5.1** | **-7.1** |
| **37** | **5280704** | **Apigetrin** | **-8.0** | **-7.6** |
| **38** | **6918391** | **Beta-Elemene** | **-7.1** | **-10.9** |
| **39** | **222284** | **Beta-sitosterol** | **-9.7** | **-12.0** |
| **40** | **10856614** | **Alpha-Selinene** | **-7.2** | **-8.7** |
| 41 | 5280443 | Apigenin | -7.5 | -6.7 |
| 42 | 5273469 | Benzyl cinnamate | -6.4 | -4.5 |
| 43 | 10104370 | Beta-bisabolene | -4.8 | -4.4 |
| 44 | 5280489 | Beta-Carotene | -6.0 | -5.3 |
| 45 | 6451618 | Alpha-Thujene | -4.1 | -5.1 |
| 46 | 15228937 | Beta-farnesene | -3.4 | -5.4 |
| 47 | 11142 | Beta-phellandrene | -3.1 | -4.1 |
| 48 | 14896 | Beta-pinene | -3.1 | -5.1 |
| 49 | 31244 | Anisaldehyde | -4.9 | -3.9 |
| 50 | 66841 | Beta-terpinene | -4.0 | -4.9 |
| 51 | 6448 | Bornyl acetate | -5.3 | -6.7 |
| 52 | 173183 | Campesterol | -6.0 | -3.6 |
| 53 | 6616 | Camphene | -5.6 | -4.6 |
| 54 | 7438 | Carveol | -2.4 | -2.4 |
| 55 | 1742210 | Caryophyllene oxide | -6.0 | -2.0 |
| 56 | 5281515 | Caryophyllene | -3.7 | -3.7 |
| 57 | 305 | Choline | -5.0 | -5.0 |
| 58 | 23663965 | Cosmosin | -1.0 | -1.0 |
| 59 | 326 | Cuminaldehyde | -1.9 | -1.9 |
| 60 | 10820 | Cuminic acid | -5.6 | -4.6 |
| 61 | 5280637 | Cynaroside | -6.9 | -5.4 |
| 62 | 3314 | Eugenol | -5.8 | -5.8 |
| 63 | 445070 | Farnesol | -4.0 | -4.0 |
| 64 | 5281522 | Isocaryophyllene | -4,9 | -4,9 |
| 65 | 22311 | Limonene | -6.7 | -7.1 |
| 66 | 6549 | Linalool | -2.5 | -2.5 |
| 67 | 5280445 | Luteolin | -6.1 | -3.4 |
| 68 | 31253 | Myrcene | -3.7 | -3.7 |
| 69 | 10582 | Myrtenol | -3.2 | -3.2 |
| 70 | 938 | Niacin | -1.7 | -1.7 |
| 71 | 445639 | Oleic acid | -3.4 | -3.4 |
| 72 | 7463 | P-cymene | -1.1 | -1.1 |
| 73 | 10247670 | Piperotol | -5.4 | -4.4 |
| 74 | 6987 | Piperitone | -1.9 | -1.9 |
| 75 | 7115 | Tannin | -6.6 | -5.0 |
| 76 | 11463 | Terpinolene | -2.1 | -2.1 |
| 77 | 6989 | Thymol | -3.2 | -3.2 |
| 78 | 442501 | Alpha-Terpineol | -4.2 | -5.2 |
| ***3. Piper Nigrum*** | | | | |
| 79 | 440917 | Limonene | -4.1 | -4.1 |
| 80 | 117443 | Cubebin | -6.9 | -4.2 |
| 81 | 442355 | Alpha-copane | -3.2 | -3.2 |
| 82 | 5280450 | Alpha-linoleic acid | -3.6 | -3.6 |
| 83 | 32735 | Beta-pinone | -2.9 | -2.9 |
| 84 | 10467 | Arachidic acid | -2.9 | -2.9 |
| 85 | 54670067 | Ascorbic acid | -3.4 | -3.4 |
| 86 | 10104370 | Beta-bisabolene | -4.8 | -4.4 |
| 87 | 5281515 | Beta-caryophyllene | -4.6 | -4.6 |
| **88** | **6918391** | **Beta-elemene** | **-7.1** | **-10.9** |
| **89** | **14985** | **Alpha tocopherol** | **-8.0** | **-10.9** |
| **90** | **28237** | **Beta-selinene** | **-7.2** | **-7.8** |
| **91** | **222284** | **Beta-sitosterol** | **-9.7** | **-12.0** |
| **92** | **14350** | **Epoxydihydrocaryophyllene** | **-7.1** | **-7.1** |
| **93** | **5280863** | **Kaempferol** | **-7.5** | **-6.4** |
| 94 | 65575 | Cedrol | -1.9 | -1.9 |
| 95 | 638011 | Citral | -4.6 | -4.6 |
| 96 | 7794 | Citronellal | -5.4 | -5.4 |
| 97 | 129700027 | Citronellal acetate | -5.8 | -5.8 |
| 98 | 92780 | Cryptone | -4.0 | -4.0 |
| 99 | 441005 | Delta cadinene | -4,9 | -4,9 |
| 100 | 518975 | Calamene | -5.0 | -5.0 |
| 101 | 119 | GABA | -2.5 | -2.5 |
| 102 | 1549026 | Geranyl acetate | -3.4 | -3.4 |
| 103 | 8438 | Heliotropine | -3.7 | -3.7 |
| 104 | 94741 | Hentriacontan-16-one | -3.2 | -3.2 |
| 105 | 12410 | Hentriacontane | -1.7 | -1.7 |
| 106 | 68345 | Hentriacontanol | -3.4 | -3.4 |
| 107 | 170833 | Isopulegol | -1.1 | -1.1 |
| 108 | 173183 | Campesterol | -6.0 | -3.6 |
| 109 | 527428 | Limonene-4-ol | -1.9 | -1.9 |
| 110 | 8294 | Linalyl acetate | -5.0 | -5.0 |
| 111 | 55255958 | Methyl caffeic acid | -2.1 | -2.1 |
| 112 | 637520 | Methyl cinnamte | -3.2 | -3.2 |
| 113 | 80790 | Methyl carvacrol | -2.3 | -2.3 |
| 114 | 11493622 | Monoterpenes | -3.4 | -3.4 |
| 115 | 4276 | Myristicin | -3.2 | -3.2 |
| 116 | 17429 | N-formylpiperidine | -2.9 | -2.9 |
| 117 | 5280343 | Quercetin | -7.0 | -2.9 |
| 118 | 25202482 | Sesquisabinene | -3.7 | -3.7 |
| 119 | 11230 | Terpinen-4-OL | -3.2 | -3.2 |
| 120 | 4462 | Ubiquinone | -2.9 | -2.9 |
| ***4. Curcuma Longa*** | | | | |
| 121 | 2758 | 1,8-CINEOLE | -4.8 | -4.8 |
| 122 | 7460 | Alpha phellandrene | -5.7 | -5.7 |
| 123 | 558221 | AR-Tumerone | -5.2 | -5.2 |
| 124 | 7462 | Alpha terpinene | -4.5 | -4.5 |
| 125 | 442501 | Alpha terpineol | -4.2 | -5.2 |
| **126** | **14985** | **Alpha tocopherol** | **-8.0** | **-6.7** |
| **127** | **6654** | **Alpha pinene** | **-6.9** | **-7.1** |
| **128** | **222284** | **Beta-sitosterol** | **-9.7** | **-12.0** |
| **129** | **444539** | **Cinnamic acid** | **-7.1** | **-7.1** |
| **130** | **69879809** | **Cyclocurcumin** | **-7.5** | **-9.1** |
| **131** | **5469424** | **Demethoxycurcumin** | **-6.9** | **-8.9** |
| **132** | **493570** | **Riboflavin** | **-7.2** | **-6.9** |
| 133 | 3033866 | Bisabolene | -3.5 | -3.5 |
| 134 | 5315469 | Bisacumol | -2.4 | -2.4 |
| 135 | 14287397 | Bisacurone | -6.1 | -2.0 |
| 136 | 64685 | Borneol | -3.7 | -3.7 |
| 137 | 689043 | Caffeic acid | -5.0 | -5.0 |
| 138 | 637429 | Calebin-A | -6.1 | -1.0 |
| 139 | 173183 | Campesterol | -6.0 | -3.6 |
| 140 | 6616 | Camphene | -5.6 | -4.6 |
| 141 | 2537 | Camphor | -5.4 | -5.4 |
| 142 | 379 | Caprylic acid | -5.8 | -5.8 |
| 143 | 26049 | CAR-3-ENE | -4.0 | -4.0 |
| 144 | 2758 | Cineole | -4,9 | -4,9 |
| 145 | 3081930 | Curzerenone | -4.3 | -4.3 |
| 146 | 92139 | Curcumene | -4.5 | -4.5 |
| 147 | 167812 | Curcumenol | -6.0 | -6.0 |
| 148 | 969516 | Curcumin | -6.7 | -6.7 |
| 149 | 14240392 | Curcumol | -6.0 | -5.4 |
| 150 | 6441391 | Curdione | -5.4 | -5.4 |
| 151 | 196216 | Curlone | -6.5 | -6.5 |
| 152 | 5280489 | Beta-carotene | -6.0 | -5.3 |
| 153 | 519764 | Beta-sesquiphellandrene | -3.9 | -3.9 |
| 154 | 10104370 | Beta-bisabolene | -4.8 | -4.4 |
| 155 | 3314 | Eugenol | -3.8 | -3.8 |
| 156 | 9548705 | Germacrene | -1.9 | -1.9 |
| 157 | 460 | Guaiacol | -5.0 | -5.0 |
| 158 | 9548703 | Guaiane | -2.1 | -2.1 |
| 159 | 22311 | Limonene | -3.2 | -3.2 |
| 160 | 6549 | Linalool | -2.3 | -2.3 |
| 161 | 938 | Niacin | -3.4 | -3.4 |
| 162 | 637542 | P-Coumaric acid | -3.2 | -3.2 |
| 163 | 7463 | P-Cymene | -2.9 | -2.9 |
| 164 | 72 | Protocatechuic acid | -2.9 | -2.9 |
| 165 | 5280343 | Quercetin | -3.7 | -3.7 |
| 166 | 5315472 | Bisdemethoxycurcumin | -6.9 | -3.6 |
| 167 | 10742 | Syringic acid | -2.9 | -2.9 |
| 168 | 17100 | Terpineol | -3.5 | -3.5 |
| 169 | 1130 | Thiamin | -3.2 | -3.2 |
| 170 | 14367555 | Turmerone | -4.3 | -4.3 |
| 171 | 101695906 | Turmeronol | -2.8 | -2.8 |
| 172 | 10955433 | Turmeronol B | -5.4 | -5.4 |
| 173 | 8468 | Vanillic acid | -5.3 | -5.3 |
| 174 | 92776 | Zingiberene | -3.2 | -3.2 |
| ***5. Allium Sativum*** | | | | |
| 175 | 133337 | 2-vinyl-1,3-dithiine | -4.9 | -4,9 |
| 176 | 444899 | Arachidonic acid | -5.6 | -5.6 |
| 177 | 94204 | 24-methylenecycloartanol | -4.5 | -4.5 |
| 178 | 5386591 | Ajoene | -6.0 | -6.0 |
| 179 | 65036 | Allicin | -6.7 | -6.7 |
| 180 | 87310 | Alliin | -5.4 | -5.4 |
| 181 | 86374 | Allixin | -5.4 | -5.4 |
| 182 | 21612418 | Allyl 2-propenethiosulfinate | -6.5 | -6.5 |
| 183 | 62434 | Allyl methyl disulfide | -4.3 | -4.3 |
| 184 | 129712276 | Allyl methyl thiosulfinate | -3.4 | -3.4 |
| 185 | 61926 | Allyl methyl trisulfide | -5.6 | -5.6 |
| 186 | 5352855 | Allylpropenyl disulfide | -3.7 | -3.7 |
| 187 | 5280934 | Alpha-Linolenic acid | -3.5 | -3.5 |
| 188 | 7460 | Alpha Phyllandrene | -2.3 | -2.3 |
| **189** | **5280443** | **Apigenin** | **-7.5** | **-6.7** |
| **190** | **10219489** | **3-vinyl-1,2-dithiin** | **-7.1** | **-7.1** |
| **191** | **198016** | **Saponin** | **-5.1** | **-10.6** |
| **192** | **5280863** | **Kaempferol** | **-7.5** | **-6.9** |
| **193** | **10717615** | **Beta chlorogenin** | **-8.4** | **-8.4** |
| **194** | **6444001** | **Xyloglucan** | **-7.1** | **-7.1** |
| **195** | **6857447** | **Beta tocopherol** | **-5.2** | **-10.5** |
| 196 | 171548 | Biotin | -3.8 | -3.8 |
| 197 | 689043 | Caffeic acid | -1.9 | -1.9 |
| 198 | 1794427 | Chlorogenic acid | -5.0 | -5.0 |
| 199 | 638011 | Citral | -2.1 | -2.1 |
| 200 | 9750 | Citrulline | -3.2 | -3.2 |
| 201 | 16590 | Diallyl disulfide | -2.3 | -2.3 |
| 202 | 23496 | Dimehtyldiselenide | -3.4 | -3.4 |
| 203 | 11648 | Dimethyl selenide | -3.2 | -3.2 |
| 204 | 866 | Endolysin | -2.9 | -2.9 |
| 205 | 445858 | Ferulic acid | -2.9 | -2.9 |
| 206 | 637566 | Geraniol | -3.7 | -3.7 |
| 207 | 5281166 | Jamonic acid | -3.2 | -3.2 |
| 208 | 801 | Auxin | -1.2 | -1.2 |
| 209 | 5280450 | Linoleic acid | -3.5 | -3.5 |
| 210 | 5280934 | Linolenic acid | -3.2 | -3.2 |
| 211 | 11005 | Myrisitic acid | -4.3 | -4.3 |
| 212 | 938 | Niacin | -2.8 | -2.8 |
| 213 | 10494 | Oleanolic acid | -5.4 | -5.4 |
| 214 | 445639 | Oleic acid | -5.3 | -5.3 |
| 215 | 6613 | Pantothenic acid | -4.7 | -4.7 |
| 216 | 441476 | Pectin | -3.4 | -3.4 |
| 217 | 19234 | Phytin | -2.7 | -2.7 |
| 218 | 5280343 | Quercetin | -4.6 | -4.6 |
| 219 | 338 | Salicyclic acid | -5.3 | -5.3 |
| 220 | 54670067 | Ascorbic acid | -1.9 | -1.9 |
| 221 | 637775 | Sinapic acid | -1.9 | -1.9 |
| 222 | 5280794 | Stigmasterol | -4.6 | -4.6 |
| 223 | 1110 | Succinic acid | -5.4 | -5.4 |
| 224 | 1123 | Taurine | -5.8 | -5.8 |
| 225 | 5570 | Trigonelline | -4.0 | -4.0 |
| 226 | 8468 | Vanillic acid | -4,9 | -4,9 |
| 227 | 11142 | Beta phyllandrene | -3.1 | -4.1 |

**Summary**

We have selected potential phytocompounds from each plants based on binding affinity values are shown in above table. The following table is summarized below;

| **Sl. No** | **Plant name** | **No. of selected compounds** |
| --- | --- | --- |
| 1 | *Zingiber offinale* | 05 |
| 2 | *Cuminum Cyminum* | 05 |
| 3 | *Piper Nigrum* | 06 |
| 4 | *Curcuma Longa* | 07 |
| 5 | *Allium Sativum* | 07 |
| **Total no. of compounds** | | **30** |

**Supplementary table 2**

**Overlapped phytocompounds from the taken five plants**

| **S.No** | **phytoconstituents** | ***Zingiberoffinale*** | ***Cuminum cyminum*** | ***Piper Nigrum*** | ***Curcuma Longa*** | ***Allium Sativum*** |
| --- | --- | --- | --- | --- | --- | --- |
|  |  | **Overlaps** | | | | |
|  | Alpha-Terpinene | Present | -- | -- | Present | -- |
|  | Alpha-Terpineol | Present | Present | -- | Present | -- |
|  | Alpha-Copaene | Present | -- | Present | -- | -- |
|  | Linolenic acid | Present | -- | -- | -- | Present |
|  | Alpha-Phellandrene | Present | Present | -- | Present | -- |
|  | Alpha-Pinene | Present | Present | -- | Present | -- |
|  | Alpha-Selinene | Present | Present | -- | -- | -- |
|  | Beta-Elemene | Present | Present | Present | -- | -- |
|  | Beta-sitosterol | Present | Present | Present | Present | -- |
|  | Kaempferol | Present | -- | Present | -- | Present |
|  | Curcumin | Present | -- | -- | Present | -- |
|  | Limonene | Present | Present | Present | Present | -- |
|  | 1,8-Cineol | Present | -- | -- | Present | -- |
|  | Apigenin | -- | Present | -- | -- | Present |
|  | Beta-bisabolene | -- | Present | Present | Present | -- |
|  | Beta-Carotene | -- | Present | -- | Present | -- |
|  | Campesterol | -- | Present | -- | Present | -- |
|  | Camphene | -- | Present | -- | Present | -- |
|  | Eugenol | -- | Present | -- | Present | -- |
|  | Linalool | -- | Present | -- | Present | -- |
|  | Niacin | -- | Present | -- | Present | Present |
|  | Oleic acid | -- | Present | -- |  | Present |
|  | P-Cymene | -- | Present | -- | Present | -- |
|  | Alpha tocopherol | -- | -- | Present | Present | -- |
|  | Quercetin | -- | -- | Present | Present | Present |
|  | Caffeic acid | -- | -- | -- | Present | Present |

**Summary:**

Among 227 phytocompounds, totally 26 phytocompounds are overlapped with taken five plants. The overlapped compounds are indicated as ‘present’ and non overlapped phytocompounds are represented as ‘—‘.
